# Study on the Molding Factors of Preparing High-Strength Laminated Bamboo Composites

**DOI:** 10.3390/ma17092042

**Published:** 2024-04-26

**Authors:** Leufouesangou Colince, Jun Qian, Jian Zhang, Chunbiao Wu, Liyuan Yu

**Affiliations:** College of Chemistry and Materials Engineering, Zhejiang A&F University, No. 666 Wusu Street, Lin’an District, Hangzhou 311300, China; cleufoue@gmail.com (L.C.); zhangjianwst@zafu.edu.cn (J.Z.); wuchunbiao123@163.com (C.W.); 15720613196@163.com (L.Y.)

**Keywords:** laminated bamboo composite, hot press molding, mechanical properties, ANOVA

## Abstract

To promote the development of the ‘Bamboo as a Substitute for Steel’ proposal, rotary cut bamboo veneers were applied to prepare a kind of high-strength laminated bamboo composite, which was achieved through the hot press molding method in this study. Orthogonal experiments of L9 (3^3^) were performed, with hot-pressing temperature, pressure, and time considered as three influencing factors. Physical properties like density and moisture content, and mechanical properties like modulus of rupture (MOR), modulus of elasticity (MOE), shear strength, and compressive strength were tested for the samples. It can be obtained from the results of range analysis and ANOVA that higher density and lower moisture content were correlated with higher mechanical strength. Within the selected range of tested factors, a hot-pressing temperature and time of 150 °C and 10 min can contribute to higher density and lower moisture content, and the combination of 150 °C and 50 MPa can produce greater mechanical strength. In the thickness direction, the laminated bamboo composites displayed a notable compressed structure.

## 1. Introduction

Bamboo belongs to the grass family and has a significantly higher growth rate than wood [1]. Various kinds of bamboo are characterized by their stems formed by a thatch, usually hollow, lignified, very fast growing, remaining one of the last plant resources that has not been massively exploited [2,3,4]. As a renewable, natural bio-composite, bamboo shows several advantages like the outstanding mechanical properties it possesses [5,6]. Given this, multiple bamboo products are commonly used in a wide range of applications like flooring, roofing, furniture, building, and other fields [7,8].

In the effort of dealing with global climate change, reducing carbon emission is urgently required. Therefore, bamboo’s remarkable mechanical properties make bamboo-based materials a good substitute for wood and steel, and quite valuable in the construction and building industry [9,10,11]. For instance, engineered bamboo materials show excellent vibration damping and vibration suppression properties due to their stiffness and damping effects, giving them wide application prospects for green low-carbon buildings [12,13].

‘Bamboo as a Substitute for Steel’ is one of the development directions of the bamboo industry in the future. The available products of ‘Bamboo as a Substitute for Steel’ include bamboo bus station, bamboo bridge, bamboo highway guardrail, and other outdoor products with high performance requirements. Additionally, bamboo nails and bamboo-based surface bearing beams have gained attention recently [11,14]. The use of a bamboo highway guardrail can not only fully utilize the bamboo’s elasticity, high toughness, and impact resistance, it can also diminish the secondary damage to the vehicle passengers [15]. According to previous research, bamboo nails can not only overcome the wood shrinkage and swelling difference between steel nails, copper nails, and jointed wood, but can also be recyclable when they are out of use without decay concentration, as black colored spots affect visual performance and have a destructive interaction with reprocessing tools [16,17,18]. Xu et al. studied the performance of bamboo nails as a novel connector for timber assemblies [18]. They found that highest MOR value of the target connected with the densified nail was 202 MPa at the density of 1.12 g/cm^3^, which was obviously greater than the un-densified nail (150 MPa).

When it comes to the above-mentioned structural and building applications, bamboo materials need to display a high strength and strength-to-weight ratio. Bamboo bundles can be joined from the width direction to form a bamboo bundle fibrillated veneer [19]. Glue-laminated bamboo is another important method to achieve the expected mechanical properties [6]. The unit is typically made up of bamboo strips, which is glued and pressed together into beams or panels. Specifically, bamboo strips can be designed to form fabricated composites with different layers. Manik et al. studied the mechanical properties of laminated bamboo composites with 3, 5, and 7 layers of bamboo strips. The bending strength was about 200–300 MPa and the bending modulus of elasticity was about 15 MPa [1]. At present, cross-laminated bamboo (CLB) or cross-laminated bamboo and timber (CLBT) panels can be used in wall or floor panel construction [20]. Chen et al. studied a new type of laminated bamboo–timber composite column with an improved compressive performance [21].

In this study, a kind of high-strength laminated bamboo composite was prepared by using rotary cut bamboo veneers and phenolic resin. The objective of the presented work is to investigate the influence of hot-pressing molding factors, namely temperature, pressure, and time, on primary physical and mechanical properties. Therefore, an orthogonal experiment of L9 (3^3^) was designed and performed. According to the relevant standards of bamboo, density, moisture content, MOR, MOE, shear strength, and compressive strength of the prepared laminated bamboo composites were tested and analyzed. Combined with SEM observations, the optimized technological process was obtained, which can provide necessary technical support to the development and research on laminated bamboo materials with superior mechanical performance.

## 2. Materials and Methods

### 2.1. Materials

**Figure 1:**
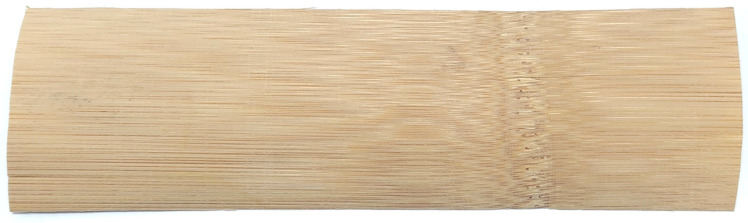
Bamboo veneer unit.

The bamboo veneer used in this study was processed by shaping and rotary cutting technology near 1/3 of the bamboo green, with a thickness of 0.2 ± 0.03 mm and an average density of 0.7 g/cm^3^. The bamboo was taken from fresh round bamboo aged 4–5 years old. The bamboo veneers were then cut into dimensions with a length of 150 mm and a width of 50 mm without obvious defects (see Figure 1).

Melamine-modified water-soluble phenolic resin was used to produce laminated bamboo composites. The solid content of the resin is about 23.5%, the viscosity is 140–150 CPS, and the total amount of coating is 190–215 g/m^2^ (liquid and permeable coating).

### 2.2. Experimental Instruments and Equipment

**Figure 2:**
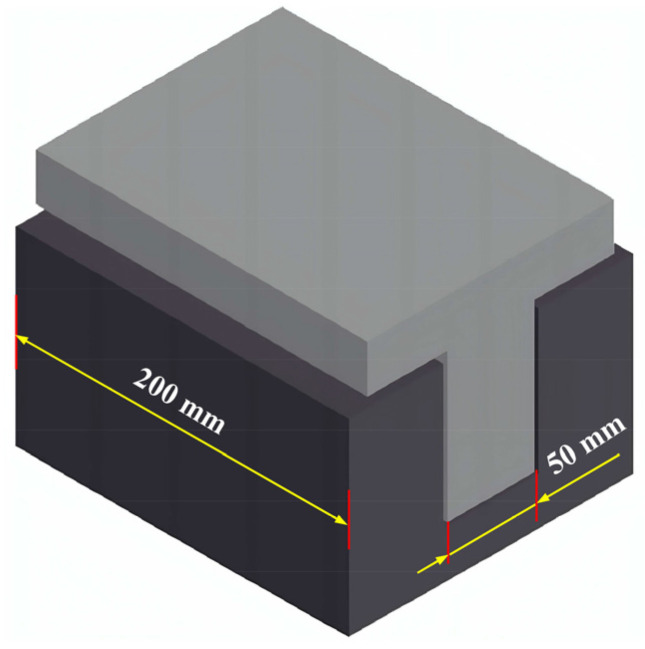
Self-made forming mold.

**Figure 3:**
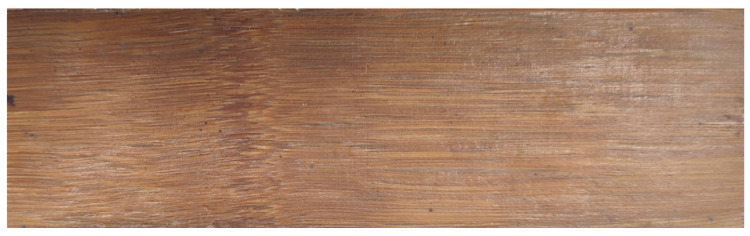
High-strength laminated bamboo composite.

The moisture content of the bamboo veneers before and after coating was controlled by using a drying oven (SEG-021, Espec, Shanghai, China). A pressure tank was self-made of stainless steel, which was used for vacuuming and high-pressure enhancer penetrating. Specific details of the pressure tank are as follows: diameter 320 mm, length 500 mm, maximum pressure 4.6 MPa, and maximum vacuum 0.08 MPa. The assembled bamboo veneer mat is placed into the grooves by using a self-made mold (see Figure 2) that is resistant to high pressure. Specific details of the mold are as follows: part one with ‘convex’ shape structure, part two with ‘concave’ shape, and groove size 50 mm × 200 mm. The mold and the assembled bamboo veneer mat were then placed into the hot press (XLB-D 500 × 500, JXR, Qingdao, China) under high pressure. The prepared laminated bamboo composite was machined into samples by a precision table circular saw (MJ 61328D, MAS, Foshan, China), as can be seen in Figure 3. The static bending strength and MOE were tested by a universal mechanical testing machine (INSTRON 5967, Norwood, MA, USA). The shear strength of the cross-section plane was tested by a self-made cutting die. A scanning electron microscope (TM3030, Hitachi, Tokyo, Japan) was used to observe the profile structure of the laminated material in the thickness direction.

### 2.3. Methods

#### 2.3.1. Experimental Methods

Preliminary experimental results suggest that temperature, pressure, and time are three key molding factors. The orthogonal test of 3 factors and 3 levels was then designed. Each case was repeated 3 times. Detailed information of the tests is listed in Table 1 and Table 2, which was determined by pre-tests. The thickness of the high-strength laminated bamboo composite was controlled by the number of bamboo veneers used (17 in this study). The prepared laminated bamboo composite was made into different testing samples for physical and mechanical properties’ measurement, including density, moisture content, average thickness, shear strength, compressive strength, MOR, and MOE, according to ISO-23478 [22]. The average value of the testing results was used for further data analysis with the help of SPSS software (IBM SPSS Statistics 27, Chicago, IL, USA).

#### 2.3.2. Resin Content Determination

Bamboo veneers with a thickness of about 0.2 mm and moisture content no more than 2% (separately placed by stainless steel grid) were put into a sealed pressure vessel and a vacuum degree of 0.06 MPa was extracted. The modified melamine-soluble phenolic resin was then penetrated. After that, the inner pressure was further increased to 2.5 MPa and kept for 110 s and then decreased to normal value. The excess resin on the bamboo veneer surface was wiped off after taking it out. Then, the penetrated veneers were oven dried to a moisture content of no more than 8%. In the self-made mold, the bamboo veneers were paved along the longitudinal direction (included angle < 5°) and put into the hot press with a temperature of 110–150 °C and a surface pressure of 20–50 MPa, lasting for 4–10 min. The resin content for the laminated bamboo composite was determined through repeated tests, which were concerned with the critical condition that the adhesive was on the leakage margin.

#### 2.3.3. Samples’ Preparation and Testing Methods of MOR and MOE

**Figure 4:**
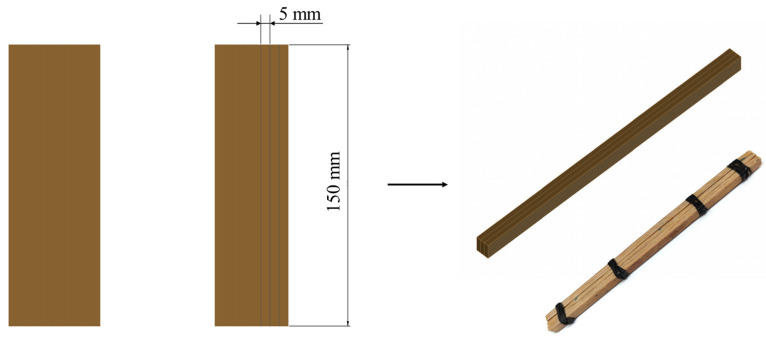
Samples for three-point bending test.

According to ISO-23478 [22], the prepared laminated bamboo composite was cut into strips with a length of 150 mm and width of 5 mm along its length direction. Since the thickness of one strip is about 3.5 mm, three of the strips were bound together with rubber bands in the thickness direction to make a testing sample. In this way, the samples could keep stable during MOR and MOE tests without disturbance (see Figure 4).

**Figure 5:**
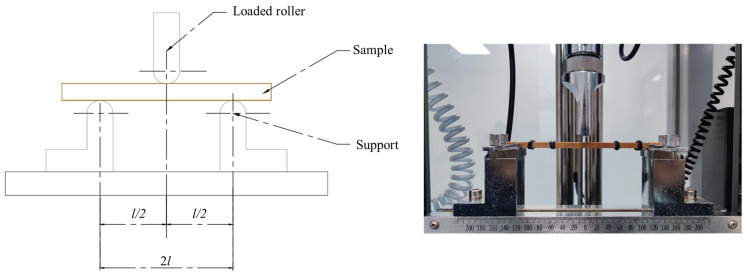
Three-point bending test for MOR and MOE measurement.

The testing samples were then placed in a condition of 20 ± 2 °C and relative humidity of 65% ± 5% to reach a local equilibrium moisture content. The measurement of MOR and MOE was conducted through a three-point bending method (see Figure 5). The distance between the two supports is 2*l*, specifically 120 mm. During the tests, the axis of the loaded roller was adjusted to be perpendicular to the samples’ central line along the length direction. In addition, the roller exerted tangential loading onto the laminated bamboo composite samples with a constant speed.

#### 2.3.4. Samples Preparation and Testing Methods of Shear Strength

**Figure 6:**
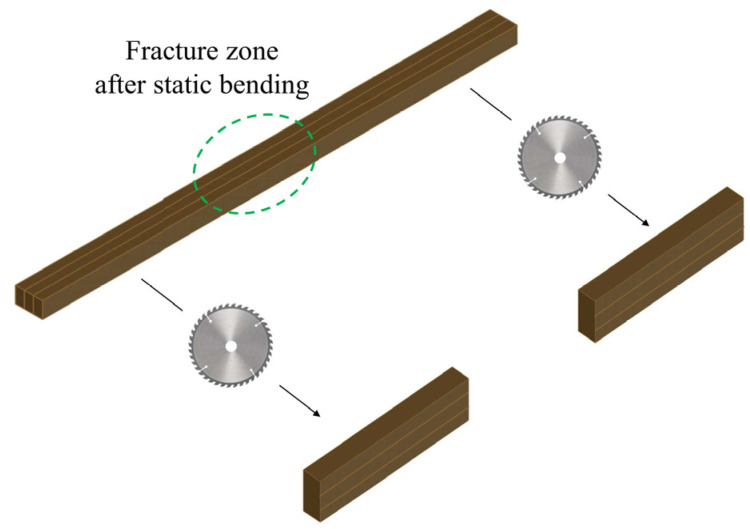
Preparation of samples for shear test.

After completing static bending tests to measure the MOR and MOE values, parts of the samples, without being damaged, were cut off by using the above-mentioned table circular saw. One of the ends was taken to shear test with a dimension of about 50 mm (L) × 5 mm (W) × 11.5 mm (H), and the other end was reserved for a compression test (see details in Section 2.3.5), as shown in Figure 6.

**Figure 7:**
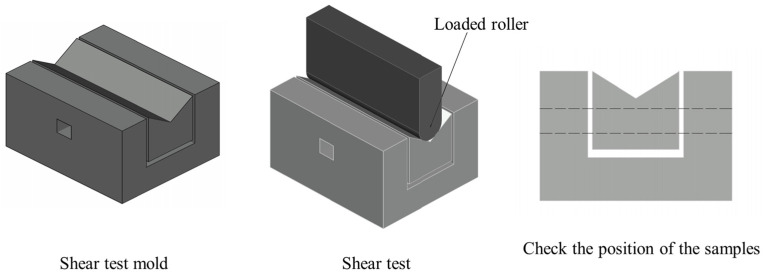
Details of the shear test.

The precise width and height of the samples were measured by a vernier caliper. Since the shear strength parallel to the bamboo fiber testing surface is two-sided, the input data should be calculated as two-sided. The sample was put into the indentation of the mold (see Figure 7), and a standardized test was carried out. During the test, the axis line of the loaded roller must be perpendicular to the center line of the mold, and the loading process should be uniform.

#### 2.3.5. Samples’ Preparation and Testing Methods of Compressive Strength

**Figure 8:**
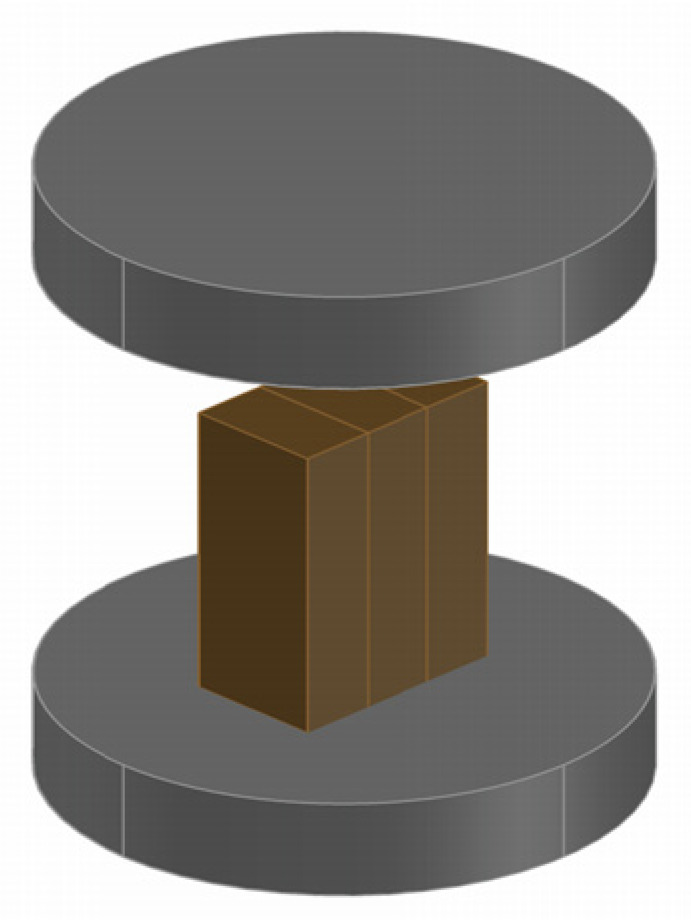
Schematic diagram of the compressive test.

The other end reserved in Section 2.3.4 was cut into 15 mm in length, and both ends were sanded to be flat. Then they were tied with rubber bands and fixed vertically and placed in the middle area of the platform, with a dimension of 15 mm (L) × 5 mm (W) × 11 mm (H) (see Figure 8). During the test of compressive strength parallel to the bamboo fiber, the pressure on the two metal discs should be evenly distributed.

## 3. Results and Discussion

**Figure 9:**
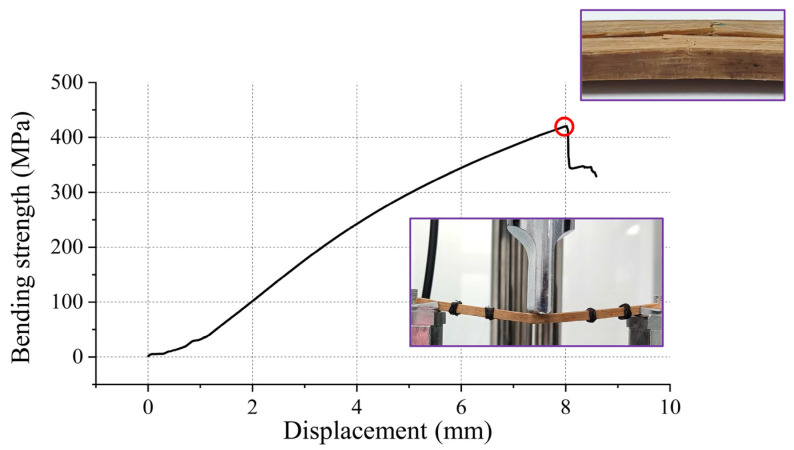
Typical strength-displacement curve and fracture of the prepared laminated bamboo composite under three-point bending loading.

The results of the designed orthogonal test L9 (3^3^) are shown in Table 3. A range analysis was conducted first for the orthogonal tests. Meanwhile, an analysis of variance (ANOVA) was used to understand a detailed visualization of the impact of the selected three factors (temperature, pressure, and time of hot pressing) influencing the fundamental physical properties of the prepared laminated bamboo composites. The comparative significance of the hot-pressing factors on the physical properties was examined based on an ANOVA analysis to obtain the more accurate optimal combination for higher strength especially. In Figure 9, the typical strength-displacement curve and fracture of the prepared laminated bamboo composite under three-point bending loading are demonstrated.

### 3.1. Density

The results of the range analysis and ANOVA about density are shown in Table 4 and Table 5. Basically, the higher density of the laminated bamboo composite could promote its strength properties, such as compressive strength parallel to grain and MOE [21,23]. In addition, density also exerts an opposite impact on the thickness expansion rate of water absorbing. Sharma et al. reported that the density of glue-laminated bamboo was 0.69 g/cm^3^ [9]. Rusch et al. reported the density of glue-laminated bamboo to be 0.77 g/cm^3^, and Appiah et al. obtained the basic density of the matured glue-laminated bamboo with 0.83 g/cm^3^ [24,25]. The testing results in this study showed that the basic density of the prepared laminated bamboo composite was generally more than 1.30 g/cm^3^, which can be primarily explained by the use of a thin bamboo veneer unit.

According to the results of the range analysis in Table 4, it can be found that the influencing order on the samples’ density is B > A > C. Specifically, the hot-pressing pressure from 20 MPa to 50 MPa has a great influence on the density change of the obtained laminated bamboo composite. In terms of higher density, the calculated optimal combination of hot-pressing factors is A_3_B_3_C_2_ and A_3_B_3_C_3_, namely 150 °C, 50 MPa, and 7 or 10 min. It can be observed from Table 5 that only hot-pressing pressure shows a significant influence on the density at a 95% confident level, while the factors of hot-pressing temperature and time are not significant even at a 90% confident level. Therefore, a convenient way to improve the laminated bamboo composite’s density is to increase the hot-pressing pressure.

**Figure 10:**
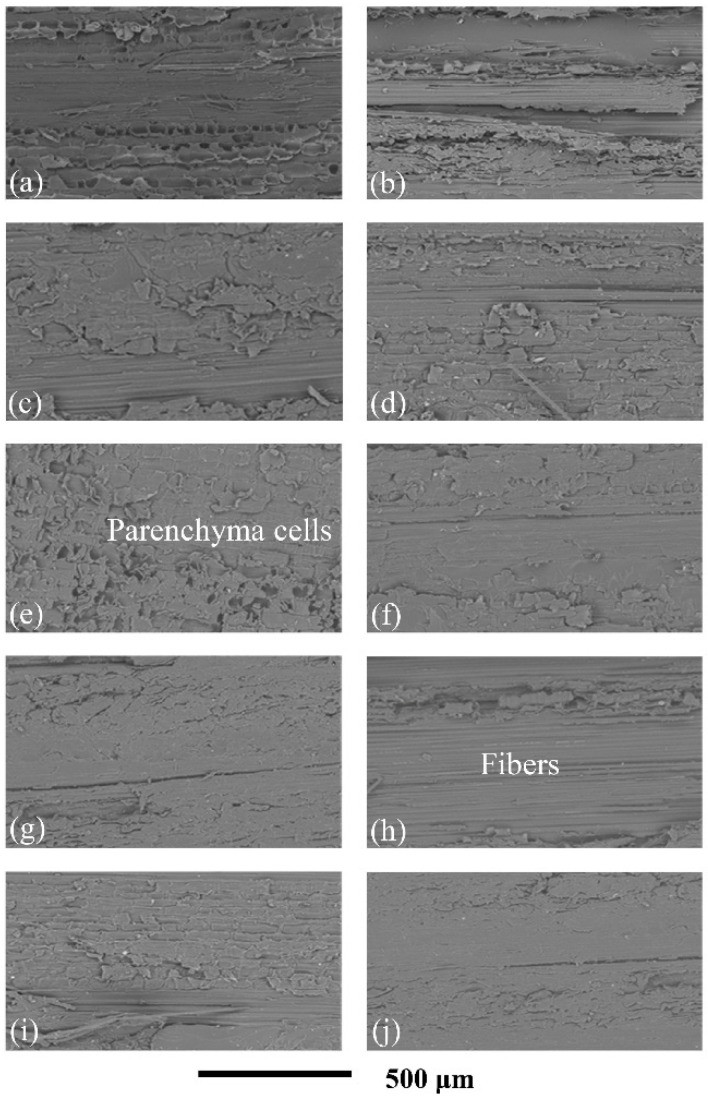
SEM images of the laminated structure in the thickness direction of the prepared high-strength laminated bamboo composite: (**a**) radial section of raw bamboo; (**b**–**j**) radial section of samples 1 to 9.

The laminated structure’s observation was conducted by using a scanning electron microscope. As can be seen from Figure 10, the laminated bamboo composites displayed a notable compressed structure in the thickness direction in comparison with raw bamboo material. The anatomical cavities primarily within vessels, parenchyma cells, and fibers collapsed during the molding process. Thus, the density of the laminated bamboo composite samples 1–9 (Figure 10b–j) was apparently greater than that of raw bamboo material.

### 3.2. MOR and MOE

A higher MOR value influences material utilization potential, especially for structural and building applications [25]. Previous related research on glue-laminated bamboo indicated that the average MOR results of glue-laminated bamboo ranged from 39 to 145 MPa [26]. The testing results in this study showed that the MOR values of the prepared laminated bamboo composite were generally greater than 400 MPa. According to the results of the range analysis for MOR in Table 6, it can be found that the influencing order on the samples’ MOR is B > A > C. Specifically, the hot-pressing pressure from 20 MPa to 50 MPa has a great impact on the MOR change of the obtained laminated bamboo composite. In terms of higher MOR, the calculated optimal combination of hot-pressing factors is A_3_B_3_C_1_, namely 150 °C, 50 MPa, and 4 min. Further, from Table 7 it can be observed that the hot-pressing pressure shows a significant influence on the MOR at a 95% confident level, while the factors of hot-pressing temperature and hot-pressing time are not significant even at a 90% confident level. Given this, greater hot-pressing pressure can be helpful to promote the curing degree of the used adhesive, and thus the MOR of the prepared composite can be improved.

MOE is an important property of glue-laminated bamboo composite that measures its stiffness or resistance to bending when subjected to stress [25]. The testing results in this study showed that the MOE in bending of the prepared laminated bamboo composite was generally bigger than 20 GPa, which was noticeably higher than the findings of Huang et al. and Sharma et al. [9,26]. According to the results of the range analysis for MOE in Table 8, it can be found that the influencing order on the samples’ MOE is B > A > C. Specifically, the hot-pressing pressure from 20 MPa to 50 MPa has a great impact on the MOE change of the obtained laminated bamboo composite. In terms of higher MOE, the calculated optimal combination of hot-pressing factors is A_2_B_3_C_3_, namely 130 °C, 50 MPa, and 7 min. Further, from Table 9 it can be observed that the hot-pressing pressure shows a significant influence on the MOE at a 95% confident level, while the factors of hot-pressing temperature and hot-pressing time are not significant even at a 90% confident level. The above results imply that the hot-pressing pressure is intensely correlated with both MOR and MOE for the prepared bamboo composite.

### 3.3. Shear Strength

The shear properties of structural bamboo materials are essential since they govern the ultimate bearing capacity of curved beams, short-span beams, and bolted joints [27]. According to the results of the range analysis for shear strength in Table 10, it can be found that the influencing order on the samples’ shear strength is B > A > C. Specifically, the hot-pressing pressure from 20 MPa to 50 MPa has a great impact on the shear strength change of the obtained laminated bamboo composite. In terms of higher shear strength, the calculated optimal combination of hot-pressing factors is A_3_B_3_C_3_, namely 150 °C, 50 MPa, and 10 min. Further, from Table 11 it can be observed that the hot-pressing pressure shows a significant influence on the shear strength at a 95% confident level, while the factors of hot-pressing temperature and hot-pressing time are not significant even at a 90% confident level. Looking at the comparison of MOR, MOE, and shear strength, the influencing order and the significance results of the selected factors are well conformed. This could further suggest that higher MOR and MOE were usually accompanied with higher shear strength, which is an assurance of material utilization potential, especially for structural applications.

### 3.4. Compressive Strength

A previous study reported that the mean compressive strength parallel to the grain of glue-laminated bamboo was about 70 MPa [9]. By contrast, the compressive strength obtained in this study was generally more than 120 MPa. According to the results of the range analysis for compressive strength in Table 12, it can be found that the influencing order on the samples’ compressive strength is B > A > C. Specifically, the hot-pressing pressure from 20 MPa to 50 MPa has a great impact on the compressive strength change of the obtained laminated bamboo composite. In terms of higher compressive strength, the calculated optimal combination of hot-pressing factors is A_3_B_3_C_3_, namely 150 °C, 50 MPa, and 10 min. Further, from Table 13 it can be observed that the hot-pressing pressure shows a significant influence on the compressive strength at a 90% confident level, while the factors of hot-pressing temperature and hot-pressing time are not significant even at a 90% confident level. Additionally, the influencing order and the significance results of the selected factors in compressive strength analysis are almost the same as the results of MOR, MOE, and shear strength. This similarity might reveal that the hot-pressing pressure is a pivotal factor in this study for the mechanical properties of the prepared laminated bamboo composite.

### 3.5. Moisture Content

The moisture content of the prepared laminated bamboo composite was generally below 7%. The lower moisture content of the laminated bamboo composite could enhance its mechanical properties and dimensional stability [28]. According to the results of the range analysis for moisture content in Table 14, it can be found that the influencing order on the samples’ moisture content is A > C > B. Specifically, the hot-pressing temperature from 110 °C to 150 °C has a great effect on the moisture content change of the obtained laminated bamboo composite. In terms of lower moisture content, the calculated optimal combination of hot-pressing factors is A_3_B_2_C_3_, namely 150 °C, 35 MPa, and 10 min. Further, from Table 15 it can be observed that hot-pressing temperature and time show a significant influence on the moisture content both at a 99% confident level, while hot-pressing pressure is not significant even at a 90% confident level.

## 4. Conclusions

When it comes to structural applications like flooring panels and jointing applications like bamboo nails, laminated bamboo composites are expected to show great mechanical properties. In this study, a kind of high-strength laminated bamboo composite was designed and prepared through a hot press molding method. The results of the range analysis and ANOVA of the performed L9 (3^3^) orthogonal experiments were carefully analyzed and discussed. The hot-pressing pressure showed a significant effect on the sample’s density. Additionally, the hot-pressing temperature and time displayed a significant influence on the sample’s moisture content. Within the selected range of tested factors, a hot-pressing temperature and time of 150 °C and 10 min can contribute to higher density and lower moisture content. MOR, MOE, shear strength, and compressive strength were tested for the samples. It can be concluded that the hot-pressing pressure exerted a significant impact on the four kinds of mechanical properties. The combination of 150 °C and 50 MPa can produce greater mechanical strength within the selected range of tested factors. The results confirmed that higher density and lower moisture content were correlated with higher mechanical strength. Owing to the slight difference in the optimal combination of MOR, MOE, and moisture content, the overall better combination of the selected influencing factors is 150 °C, 50 MPa, and 10 min in this study.

## Figures and Tables

**Table 1:** Testing factors and levels.

A—Temperature (°C)	B—Pressure (MPa)	C—Time (min)
110	20	4
130	35	7
150	50	10

**Table 2:** Arrangement of orthogonal test L9 (3^3^).

Trial No.	Temperature (°C)	Pressure (MPa)	Time (min)
A1	110	20	4
A2	110	35	7
A3	110	50	10
B1	130	20	7
B2	130	35	10
B3	130	50	4
C1	150	20	10
C2	150	35	4
C3	150	50	7

**Table 3:** Physical and mechanical testing results of L9 (3^3^).

Trial No.	A (°C)	B (MPa)	C (min)	Density (g/cm^3^)	MOR (MPa)	MOE (MPa)	Shear Strength (MPa)	Compressive Strength (MPa)	Moisture Content (%)
A_1_	1 (110)	1 (20)	1 (4)	1.23	382.10	19,214.52	58.47	105.68	7.71
A_2_	1	2 (35)	2 (7)	1.32	418.55	22,638.50	63.47	128.04	7.13
A_3_	1	3 (50)	3 (10)	1.37	491.36	25,688.03	69.41	143.78	6.82
B_1_	2 (130)	1	2	1.30	404.50	21,602.32	62.24	126.40	7.18
B_2_	2	2	3	1.34	422.91	28,468.46	68.23	135.79	6.53
B_3_	2	3	1	1.35	494.72	29,220.38	69.86	144.31	7.41
C_1_	3 (150)	1	3	1.30	433.07	24,065.81	63.22	134.69	5.76
C_2_	3	2	1	1.35	480.22	25,100.20	69.40	136.82	6.45
C_3_	3	3	2	1.39	509.99	28,534.23	74.90	150.30	6.24

**Table 4:** Results of range analysis for density.

Trial No.	A (°C)	B (MPa)	C (min)	Description
*k*_1_	1.31	1.28	1.31	Influencing order: B > A > C Optimal combination: A_3_B_3_C_2_ and A_3_B_3_C_3_
*k*_2_	1.33	1.34	1.34	
*k*_3_	1.35	1.37	1.34	
*r*	0.04	0.09	0.03	

**Table 5:** Results of ANOVA for density.

Factor	DF	Mean Square	*F* Ratio	*F*_0.01_	*F*_0.05_	*F*_0.10_	Significance
A	2	0.001211	3.516	99.00	19.00	9.00	*Not significant*
B	2	0.006711	19.484	99.00	19.00	9.00	*Significant at 95% confident level*
C	2	0.000711	2.065	99.00	19.00	9.00	*Not significant*
Error	2	0.000344	-	-	-	-	-

**Table 6:** Results of range analysis for MOR.

Trial No.	A (°C)	B (MPa)	C (min)	Description
*k*_1_	430.67	406.56	452.35	Influencing order: B > A > C Optimal combination: A_3_B_3_C_1_
*k*_2_	440.71	440.56	444.35	
*k*_3_	474.43	498.69	449.11	
*r*	43.76	92.13	8.00	

**Table 7:** Results of ANOVA for MOR.

Factor	DF	Mean Square	*F* Ratio	*F*_0.01_	*F*_0.05_	*F*_0.10_	Significance
A	2	1576.130544	5.068	99.00	19.00	9.00	*Not significant*
B	2	6511.937344	20.941	99.00	19.00	9.00	*Significant at 95% confident level*
C	2	48.587778	0.156	99.00	19.00	9.00	*Not significant*
Error	2	310.968311	-	-	-	-	-

**Table 8:** Results of range analysis for MOE.

Trial No.	A (°C)	B (MPa)	C (min)	Description
*k*_1_	22,513.68	21,627.55	24,511.70	Influencing order: B > A > C Optimal combination: A_2_B_3_C_3_
*k*_2_	26,433.39	25,403.39	24,258.35	
*k*_3_	25,900.08	27,814.21	26,074.10	
*r*	3919.71	6186.66	1815.75	

**Table 9:** Results of ANOVA for MOE.

Factor	DF	Mean Square	*F* Ratio	*F*_0.01_	*F*_0.05_	*F*_0.10_	Significance
A	2	1,354,447,476	8.957	99.00	19.00	9.00	*Not significant*
B	2	2,916,997,751	19.291	99.00	19.00	9.00	*Significant at 95% confident level*
C	2	290,105,308	1.919	99.00	19.00	9.00	*Not significant*
Error	2	151,218,147	-	-	-	-	-

**Table 10:** Results of range analysis for shear strength.

Trial No.	A (°C)	B (MPa)	C (min)	Description
*k*_1_	63.78	61.31	65.91	Influencing order: B > A > C Optimal combination: A_3_B_3_C_3_
*k*_2_	66.78	67.03	66.87	
*k*_3_	69.17	71.39	66.95	
*r*	5.39	10.08	1.04	

**Table 11:** Results of ANOVA for shear strength.

Factor	DF	Mean Square	*F* Ratio	*F*_0.01_	*F*_0.05_	*F*_0.10_	Significance
A	2	21.878078	8.551	99.00	19.00	9.00	*Not significant*
B	2	76.671744	29.966	99.00	19.00	9.00	*Significant at 95% confident level*
C	2	1.008544	0.394	99.00	19.00	9.00	*Not significant*
Error	2	2.558611	-	-	-	-	-

**Table 12:** Results of range analysis for compressive strength.

Trial No.	A (°C)	B (MPa)	C (min)	Description
*k*_1_	125.83	122.26	128.94	Influencing order: B > A > C Optimal combination: A_3_B_3_C_3_
*k*_2_	135.50	133.55	134.91	
*k*_3_	140.60	146.13	138.09	
*r*	14.77	23.87	9.15	

**Table 13:** Results of ANOVA for compressive strength.

Factor	DF	Mean Square	*F* Ratio	*F*_0.01_	*F*_0.05_	*F*_0.10_	Significance
A	2	168.820678	6.533	99.00	19.00	9.00	*Not significant*
B	2	427.865911	16.558	99.00	19.00	9.00	*Significant at 90% confident level*
C	2	64.756544	2.506	99.00	19.00	9.00	*Not significant*
Error	2	25.840411	-	-	-	-	-

**Table 14:** Results of range analysis for moisture content.

Trial No.	A (°C)	B (MPa)	C (min)	Description
*k*_1_	7.22	6.88	7.19	Influencing order: A > C > B Optimal combination: A_3_B_2_C_3_
*k*_2_	7.04	6.70	6.85	
*k*_3_	6.15	6.82	6.37	
*r*	1.07	0.18	0.82	

**Table 15:** Results of ANOVA for moisture content.

Factor	DF	Mean Square	*F* Ratio	*F*_0.01_	*F*_0.05_	*F*_0.10_	Significance
A	2	0.984700	317.645	99.00	19.00	9.00	*Significant at 99% confident level*
B	2	0.025200	8.129	99.00	19.00	9.00	*Not significant*
C	2	0.509200	164.258	99.00	19.00	9.00	*Significant at 99% confident level*
Error	2	0.003100	-	-	-	-	-

## Data Availability

The data are contained within the article.

## References

[1] Manik P., Samuel S., Tuswan T., Jokosisworo S., Nadapdap R.K. (2022). Mechanical properties of laminated bamboo composite as a sustainable green material for fishing vessel: Correlation of layer configuration in various mechanical tests. J. Mech. Behav. Mater..

[2] Tokoro R., Vu D.M., Okubo K., Tanaka T., Fujii T., Fujiura T. (2008). How to improve mechanical properties of polylactic acid with bamboo fibers. J. Mater. Sci..

[3] Mishra G., Giri K., Panday S., Kumar R., Bisht N.S. (2014). Bamboo: Potential resource for eco-restoration of degraded lands. J. Biol. Earth Sci..

[4] Singnar P., Das M.C., Sileshi G.W., Brahma B., Nath A.J., Das A.K. (2017). Allometric scaling, biomass accumulation and carbon stocks in different aged stands of thin-walled bamboos Schizostachyum dullooa, Pseudostachyum polymorphum and Melocanna baccifera. For. Ecol. Manag..

[5] Yu Y., Wang H., Lu F., Tian G., Lin J. (2014). Bamboo fibers for composite applications: A mechanical and morphological investigation. J. Mater. Sci..

[6] Akinyemi B.A., Omoniyi T.E. (2020). Effect of experimental wet and dry cycles on bamboo fibre reinforced acrylic polymer modified cement composites. J. Mech. Behav. Mater..

[7] Abdul Khalil H.P.S., Bhat I.U.H., Jawaid M., Zaidon A., Hermawan D., Hadi Y.S. (2012). Bamboo fibre reinforced biocomposites: A review. Mater. Des..

[8] Liu X., Smith G.D., Jiang Z., Bock M.C.D., Boeck F., Frith O., Gatóo A., Liu K., Mulligan H., Semple K.E. (2015). Nomenclature for Engineered Bamboo. BioRes.

[9] Sharma B., Gatóo A., Bock M., Ramage M. (2015). Engineered bamboo for structural applications. Constr. Build. Mater..

[10] Huang Y., Ji Y., Yu W. (2019). Development of bamboo scrimber: A literature review. J. Wood Sci..

[11] Chen G., Yu Y., Li X., He B. (2020). Mechanical behavior of laminated bamboo lumber for structural application: An experimental investigation. Eur. J. Wood Prod..

[12] Huang Z., Sun Y., Musso F. (2017). Assessment on bamboo scrimber as a substitute for timber in building envelope in tropical and humid subtropical climate zones—Part 2 performance in building envelope. IOP Conf. Ser. Mater. Sci. Eng..

[13] Ufodike C.O., Eze V.O., Ahmed M.F., Oluwalowo A., Park J.G., Liang Z., Wang H. (2020). Investigation of molecular and supramolecular assemblies of cellulose and lignin of lignocellulosic materials by spectroscopy and thermal analysis. Int. J. Biol. Macromol..

[14] Penellum M., Sharma B., Shah D.U., Foster R.M., Ramage M.H. (2018). Relationship of structure and stiffness in laminated bamboo composites. Constr. Build. Mater..

[15] Md Tahir P., Lee S.H., Osman Al-Edrus S.S., Uyup M.K.A. (2023). Multifaceted Bamboo.

[16] Korte H., Koch G., Krause K.C., Koddenberg T., Siemers S. (2018). Wood nails to fix softwoods: Characterization of structural deformation and lignin modification. Eur. J. Wood Prod..

[17] Derikvand M., Jiao H., Kotlarewski N., Lee M., Chan A., Nolan G. (2019). Bending performance of nail-laminated timber constructed of fast-grown plantation eucalypt. Eur. J. Wood Prod..

[18] Xu Y., Dong Z., Jia C., Wang Z., Lu X. (2021). Bamboo Nail: A Novel Connector for Timber Assemblies. J. Renew. Mater..

[19] Liu H., Jiang Z., Sun Z., Yan Y., Cai Z., Zhang X. (2017). Impact performance of two bamboo-based laminated composites. Eur. J. Wood Prod..

[20] Xiao Y., Cai H., Dong S.Y. (2021). A Pilot Study on Cross-Laminated Bamboo and Timber Beams. J. Struct. Eng..

[21] Chen S., Wei Y., Wang G., Zhao K., Ding M. (2023). Mechanical behavior of laminated bamboo–timber composite columns under axial compression. Archiv. Civ. Mech. Eng..

[22] (2022). Bamboo structures—Engineered Bamboo Products—Test Methods for Determination of Physical and Mechanical Properties.

[23] Yang D., Li H., Lorenzo R., Yuan C., Hong C., Chen Y. (2023). Basic Mechanical Properties of Laminated Flattened-Bamboo Composite: An Experimental and Parametric Investigation. J. Mater. Civ. Eng..

[24] Rusch F., Ceolin G.B., Hillig É. (2019). Morphology, density and dimensions of bamboo fibers: A bibliographical compilation. Pesqui. Agropecu. Trop..

[25] Appiah-Kubi E., Awotwe-Mensah M., Jobson Mitchual S. (2023). Assessment of physical and mechanical properties of juvenile and matured Bambusa vulgaris glue-laminated bamboo for structural applications in Ghana. Sustain. Struct..

[26] Dongsheng H., Aiping Z., Yuling B. (2013). Experimental and analytical study on the nonlinear bending of parallel strand bamboo beams. Constr. Build. Mater..

[27] Li Z., Xia M.K., Shi J.J., Wang R. (2022). Shear properties of composite cross-laminated bamboo panels. Eur. J. Wood Prod..

[28] Chen S., Wei Y., Zhu J., Lin Y., Du H. (2023). Experimental investigation of the shear performance of bamboo scrimber beams reinforced with bamboo pins. Constr. Build. Mater..

