# Correction: Colince et al. Study on the Molding Factors of Preparing High-Strength Laminated Bamboo Composites. *Materials* 2024, *17*, 2042

**DOI:** 10.3390/ma19071296

**Published:** 2026-03-25

**Authors:** Leufouesangou Colince, Jun Qian, Jian Zhang, Chunbiao Wu, Liyuan Yu

**Affiliations:** College of Chemistry and Materials Engineering, Zhejiang A&F University, No. 666 Wusu Street, Lin’an District, Hangzhou 311300, China; cleufoue@gmail.com (L.C.); zhangjianwst@zafu.edu.cn (J.Z.); wuchunbiao123@163.com (C.W.); 15720613196@163.com (L.Y.)

In the original publication [1], there was an overlap in Figure 10 as published. The corrected Figure 10 appears below. The authors state that the scientific conclusions are unaffected. This correction was approved by the Academic Editor. The original publication has also been updated.

## Figures and Tables

**Figure 10:**
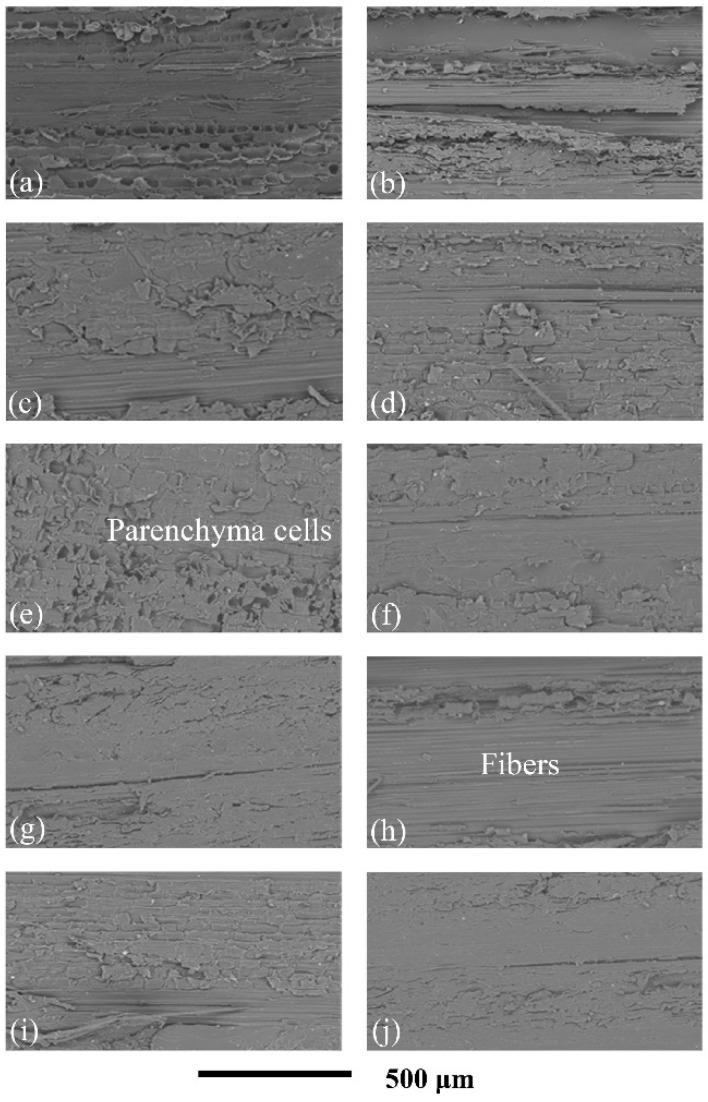
SEM images of the laminated structure in the thickness direction of the prepared high-strength laminated bamboo composite: (**a**) radial section of raw bamboo; (**b**–**j**) radial section of samples 1 to 9.
